# “Discussion or silent accompaniment: a grounded theory study about voluntary stopping of eating and drinking in Switzerland”

**DOI:** 10.1186/s12904-022-00941-4

**Published:** 2022-05-24

**Authors:** Sabrina Stängle, André Fringer

**Affiliations:** 1 Institute of Nursing, ZHAW School of Health Professions, Katharina-Sulzer-Platz 9, 8401 Winterthur, Switzerland; 2 Department of Nursing Science, Witten/Herdecke University Faculty of Health, Stockumer Strasse 12, 58453 Witten, Germany

**Keywords:** Counsellors, Ethicists, Focus group interviews, Grounded theory, Health care professionals, Relatives, Voluntary stopping of eating and drinking

## Abstract

**Background:**

Voluntary stopping of eating and drinking as an option to end life prematurely is gaining international attention, and health care professionals are increasingly confronted with the wish to die through voluntary stopping of eating and drinking by individuals. While to date, there are no guidelines in Switzerland to orient professional support, it is of interest how professionals and other people involved react to the situation. The aim of this qualitative study was to explore how health care professionals in Switzerland accompany individuals during voluntary stopping of eating and drinking and to analyze this decision-making process.

**Methods:**

Charmaz's grounded theory constructivist methodology uses guidelines for systematic, theory-driven data analysis underpinned by a pragmatic philosophical perspective. Data were collected in autumn 2016 as part of a regional palliative care conference on voluntary stopping of eating and drinking. All participants of the expert meeting (*N* = 50, including nurses, counsellors, ethicists, medical doctors, politicians, volunteers, and relatives) were invited to the focus group interviews, of which *N* = 47 participated. We conducted five focus group interviews, each lasting one hour.

**Results:**

The results showed that the accompaniment of those willing to die during voluntary stopping of eating and drinking was either discussed and cleared with one another or was unspoken and silently accompanied.

**Conclusions:**

The demands of participants for more knowledge must be heeded, and there is also a need for systematic instructions on how to proceed in the case of voluntary stopping of eating and drinking support and what needs to be considered.

## Background

Voluntary stopping of eating and drinking (VSED) is an option to die prematurely [1–5]. The international literature shows that VSED is being increasingly recognized as a viable option for hastening death in the context of end-of-life care [6–13]. Surveys among health care professionals (HCP) show that they accompany individuals internationally on this path and that it cannot be considered an isolated case [1, 6, 10, 12, 14, 15]. According to calculations from the Netherlands and Switzerland, 0.4% to 2.1% of all deaths per year are associated with VSED [2, 16–19].

In Switzerland, the Swiss Academy of Medical Sciences already perceived VSED as an option for end-of-life decisions and mentioned it when updating its guideline “Management of Death and Dying” in 2018 [20]. This guideline does not describe any instructions for handling the issue but mentions the existing possibilities of terminal care in Switzerland and their anchoring in the health care system and their acceptance. The decision for or against accompanying an individual is left to the HCP themselves [20]. VSED is described as a controversial action [21, 22]. It is controversial because VSED can be demanded at different times and with varying conditions in the life of the individual willing to die, leading to different decisions [20]. The following two cases differ mainly in terms of the time of the decision on the VSED. In the first case, an individual who already has a shortened life expectancy wants to shorten the dying process by refusing food and fluid. In the second case, an individual without a life threatening/limiting illness intends to choose the path of VSED to end life prematurely [20, 23]. The literature shows that the life expectancy of individuals before they VSED may be limited to a few days or weeks or may be more than a year [9, 12, 15]. This background and other factors (the presence of a disease [15, 24], the age of the individual [25], the fear of doing something forbidden[25, 26]) influence the decision of HCP and relatives to accompany the individual willing to die or to refuse to accompany the individual. Specifically, palliative care experts are mentored in assisting individuals at the end of life and, consequently, are a point of contact for individuals to discuss the desire to die through VSED. Accompaniment does not mean support of the individual in dying [27–29], but rather it means giving counseling on the course of the disease and any complications [5, 30, 31], symptom management [13, 32, 33], oral care [1, 25, 34], support in mobilization[30, 35] and being there for the individual [25, 36]. Regardless of this, the individual's ability to judge is a prerequisite for VSED [1, 13, 26, 30]. Voluntariness in the “V” of VSED refers to the ability to judge one's own destiny and to bear and understand the consequences for one's own actions [11, 37–39]. If an individual is not able to judge, or only to a limited extent, it must be thoroughly clarified whether the refusal of food and fluid is following the will of the individual or whether there are other reasons [40]. Here, too, the controversy is very clear. While all questions and uncertainties can be clarified with an individual capable of judgment, this is difficult to answer with an individual who is not capable or is partially incapable of using judgment. Another difficulty is that not all individuals openly communicate their desire to die. Some cannot or do not want to say so aloud, so that the HCP have to perceive the signs [41]. According to the Swiss Academy of Medical Sciences, whether an individual may be accompanied during VSED can only be decided in the individual case by the HCP themselves [20]. The findings of a single case study show that even within a single case, HCP tend to react very differently [25]. Instructions or guidelines for HCP on the decision-making process at VSED currently do not exist in Switzerland. It is also unknown how HCP, relatives and other people involved deal with the individuals willing to die.

In individuals whose life and quality of life are restricted by unbearable suffering, the desire to end their lives prematurely can mature [1, 9]. As in others, many individuals at this stage of life have the desire to make self-determined and autonomous decisions and look for solutions to change their situation [42]. The decision to choose VSED is one of them [1, 23]. Accompanying an individual during VSED is not prohibited in Switzerland, but it is also not regulated [43], comparable to the United States of America [11, 44], Canada[45, 46] and Germany [47, 48]. Although individuals and their relatives ask HCP for advice and support, they lack clear guidelines. As a result, all those involved in the process offer very individual advice and/or support [20, 23].

In addition to the lack of a guideline, there is also the added difficulty that the individuals who choose VSED are very uneven. The average age during VSED is over 80 years for most individuals (48–75%) [2, 9, 49]. Women (38–63%) and men (37–62%) decide to VSED with similar frequency [2, 12, 49]. Some individuals have underlying diseases that are relevant for the decision to VSED. It is assumed that age is to be considered a confounder since the chance of becoming ill increases with increasing age [12, 50]. The most common reasons for VSED are listed according to the biopsychosocial-spiritual model[51, 52] and can be causative alone or in combination [27]. A proportion (24–54%) of individuals have no serious diseases other than age-related frailty [2, 9, 12, 49]. In individuals without severe illnesses, the most common reasons for deciding to VSED are:

- Biological: Besides – or due to – their underlying disease, if any are present, those yearning to die suffer from a reduced quality of life [24]; pain [9]; and reduced general condition [34].
- Psychological: Feeling of loneliness [9]; and fear of (increasing) dependency [38].
- Social: Restricted mobility [25]; and increasing dependency; and social isolation [53].
- Spiritual: The desire for self-determination, autonomy, and control in dying [1]; life fatigue [13]; and senselessness of life [54].

A few reasons lead individuals to express their wish to die by VSED. This desire to die affects HCP in direct care, relatives who are emotionally involved, pastors who discuss the mental and spiritual aspects with the individual willing to die, volunteers who are mainly there and talk to the individual, etc. In other words, this group includes all people who are involved in the care and accompaniment of the individual. For reasons of readability, the term HCP will be used in the further course of this work, whereby all groups of persons mentioned are meant. With the expressed desire to die, the HCP are in the decision-making process. Will I or will I not support the individual on their way? HCP have the task of proposing alternatives to the individual willing to die.

## Methods

### Aims

This study aimed to explore how HCP in Switzerland accompany individuals during VSED and to analyze this decision-making process.

### Grounded theory methodology

We implemented a grounded theory approach, which is oriented in relation to Bryant and Charmaz[55] that supports the inductive, emergent and constant comparative approach[56] as needed to achieve the goal of this study. This qualitative methodology is appropriate to explore the experiences and meanings of HCP in the context of the decision-making processes of an individual wishing to die through VSED [55]. Constructivist researchers acknowledge that data, analyses and methodological strategies are constructed and take into account the research context and the perspectives of researchers in their interpretations [55]. The grounded theory was underpinned by a pragmatic philosophical perspective, which assumes that our knowledge is developed through our actions and interactions, which are shaped and developed by our social environment [57, 58]. Since the phenomenon under investigation in this study was little known internationally at the time of the investigation and had not been researched in Switzerland until then, the recruitment of participants was challenging, which is why theoretical sampling as an instrument of theoretical saturation could not be implemented in this research. This study is a research strand integrated in a convergent mixed method [59], which was pre-recorded in a study protocol.[60] Since at this stage the mixed-methods study could already be published [58], there are some overlaps in the description of the method. However, the method had to be greatly shortened in mixed-methods article, so that the same contents had to be taken up in this paper, but described in more detail for better comprehensibility.

### Researcher characteristics and reflexivity

The first author, a junior nursing scientist, was significantly supported and instructed by the long-standing and experienced qualitative nursing scientists (second author). The authors have familiarized themselves with the current state of knowledge prior to the interviews; none of the authors had personal experience with an individual willing to die during the VSED but have had numerous conversations with individuals who want to go this path, or with relatives and HCP who have accompanied an individual during VSED.

### Sampling and participants

We wanted to ensure that all HCP in advising and accompanying an individual during VSED were included in the study [61, 62]. Due to the difficulty of reaching HCP dealing with a controversial topic[21, 29, 31, 63, 64] and an opportunity to include a broad array of participants, an official event about VSED in the research used maximum variation as the sampling strategy and collected all interviews at one time. For this reason, an regional palliative care conference on VSED organized by “Palliativ Zug” (www.palliativ-zug.ch) also included the “piggyback” focus groups [62]. HCP and providers of all palliative services in the German-speaking canton Zug in Switzerland were invited by the cantonal palliative care association, which organizes annual public events to bring together members and interested parties on a specific topic. In autumn 2016, the topic of VSED was announced, to which the last author was invited for a lecture. We asked the initiators to use this rare opportunity to include the participants with their different professional and personal backgrounds and interest in VSED for maximum variation sampling. Already in the invitation to the event, the participants were informed that, after the lecture on the topic, focus group interviews were planned, to which all were cordially invited. Of the 50 participants in the event, *n* = 47 participants decided to participate in the focus groups, which was recorded in a written declaration of consent. Three participants did not take part in the focus groups without giving any reason and left the event [58]. The participants’ characteristics were collected using a one-page written questionnaire and are shown in Table 1.

**Table 1:** Participants’ characteristics

Participants’ characteristics in the focus groups	Mean (SD) Range	Absolute (relative %) frequencies
Total participants		47
Professional background		
(N) Nurse		17 (36.2%)
(M) Nursing manager		10 (21.3%)
(C) Counsellor (Counseling centers for age, cancer, etc.)		8 (17.0%)
(E) Ethicists (research associates, church council, pastor)		5 (10.6%)
(D) Medical doctor		2 (4.3%)
(P) Politician (local council)		2 (4.3%)
(V) Volunteer (in palliative care)		2 (4.3%)
(R) Relative		1 (2.1%)
Age (years) (missing: 8)	49 (11) 25–64	
Sex (missing: 7)		
female		35 (87.5%)
male		5 (12.5%)
Familiarity with the topic VSED (missing: 8)		
familiar/ accompanied several VSED cases		10 (25.6%)
somewhat familiar/ accompanied one VSED case		22 (56.4%)
unfamiliar/ not yet accompanied a VSED case		7 (17.9%)

### Data collection

Interview guidelines were developed for managing and structuring the focus group interviews[65] for orientation reasons and to support the moderators (see Table 2). From the beginning, however, it was emphasized that one was allowed to deviate from the interview guide at any time, so that the greatest possible diversity of knowledge and insights about this little empirically researched topic could be experienced. This was also done so intensively that the interview guide received virtually no attention. Five focus group interviews were conducted and – with the consent of the participants – digitally recorded and transcribed verbatim. We aimed to explore the experiences, attitudes and stances within the group about VSED [66], taking into account the participants' interactions and group dynamics, which is why we chose the focus group interview [62, 67]. Four focus groups consisted of 9 participants one group consisted of 11 participants [62, 66, 68]. All focus group interviews took place in parallel in a large hall each at a separate table [61]. The interviews of the focus group were conducted by very empathetic moderators with in-depth knowledge of the VSED, qualitative research and focus groups. Each interview lasted 60 min. Field notes were taken during the discussion and after the focus groups have been conducted. An assistant moderator installed the audio devices, took care of the environment (volume, drinks, etc.) and took notes. Finally, all moderators were asked to report the most important findings from their discussion in the plenum.

**Table 2:** Interview guide

All of you have deliberately registered for this expert meeting about VSED. What is your connection to this topic? What has aroused your interest? Have you been asked by your employer to participate?
Have you ever been asked by a person willing to die whether you would accompany them during VSED? Please share your reactions and feelings and those of the other participants Has the desire always been clearly expressed? If not, how did you recognize the intention? Were alternatives discussed? How did the decision-making process happen?
Have you already accompanied a person willing to die during VSED? Please tell us about your experiences What agreements were made between the HCP? How did the communication between you take place? How did you deal with hurdles or difficulties? What were your fears and worries? Who takes what role in the accompaniment? Did you know what to expect? What surprised you? In retrospect, what would you have needed in advance or during that time?

### Data analysis

The data were inductively analyzed through a modified ground theory approach, designed by Bryant and Charmaz [69], that uses guidelines for systematic, theory-driven data analysis. The topics were derived not in advance but from the data. Once the topics had been defined, they were sorted according to their chronological order. Data coding and memo writing was done using MAXQDA (Analytics Pro 2018).

The first author coded the data according to the comparative method after reading each interview several times, i.e., the data were selected, separated, and sorted and coded inductively line by line (initial coding). The process was assisted by an experienced qualitative researcher (last author). Next, we searched for relationships between frequently initial codes or codes with high significance and, after connecting them, built-up categories (selective coding). Through theoretical integration, we identified one core category and could define to subcategories strategies that relate to the core category and made this visible throughout a figure (theoretical coding).

### Trustworthiness

To obtain an accurate and truthful depiction of the participants' attitudes about VSED, the participants were given a definition about the VSED before the interviews and reads as follows: *“The VSED is a conscious decision taken by a competent, capacitated person willing to end life prematurely.”* This was the only way to ensure that everyone's understanding of the phenomenon was clear and that the statements were concretely related to the phenomenon (Credibility). Through core and subcategories, new insights about the public influence on VSED and about different ways HCP focus when being confronted with the topic (Originality). A senior nursing scientist (last author) reviewed the results of the junior nursing scientist (first author) to check the identified topics and descriptors from his own perspective. All new topics and descriptors highlighted by the last author were accepted and compared with my own analysis of the entire focus group data. Notes were made to document the steps in the analysis. Meetings were also held to examine the processes in which data were analyzed and interpreted (Resonance). The results of this study are very helpful for healthcare professionals. It shows that the topic is relevant in the healthcare setting and that professional handling is necessary to develop a systematic approach to take care of individuals who wish to hasten death by VSED. The developed knowledge can help professionals become aware of the topic and be prepared before being confronted by an individual (Usefulness) [70].

### Ethical considerations

The responsible institutional review board of the Greater Region of Eastern Switzerland (EKOS 17/083) approved this study. All participants were informed of the objective and design of the study and written consent was received from the participants for interviews, and they were free to leave the focus group if they wished.

## Results

**Fig. 1:**
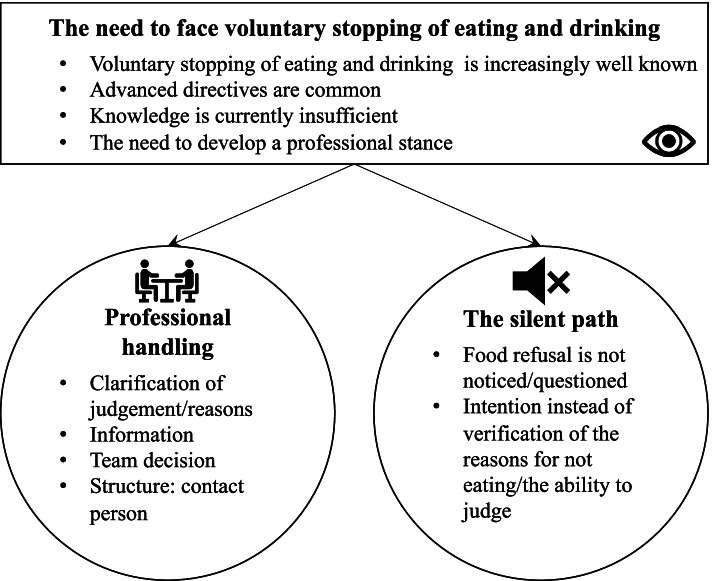
Core findings about the meaning of VSED patterns of behavior of HCP

During the analysis, the core concept “the need to face VSED” emerged, which describes the meaning of the phenomenon by the HCP, and two main categories as behavior patterns were discovered that describe the decision-making process in the case of VSED accompaniment: discussion, and the silent path (Fig. 1).

### The need to face VSED

Participants pointed out that VSED is a phenomenon that is becoming increasingly important. Death as a subject matter has been ignored for decades, but society is beginning to talk openly about death. Several participants have already been approached about VSED in their professional and private lives. In fact, the way someone desires to spend the rest of his or her days is emerging as an increasingly relevant topic. HCP accept and demand the wish of many individuals to make self-determined decisions in health matters. Furthermore, they support individuals responding to their needs and desires at the end of their lives and including them in their advance directive. The participants argue that although medical progress has significantly influenced the lives of many individuals and has led to a significant improvement in (survival) life and quality of life, not all unbearable conditions of suffering can be adequately treated today. This can lead individuals to decide against further therapies or to withdraw them. Participants state that the withdrawal of therapy is not necessarily associated with the end of suffering. Surviving and living on, despite unbearable conditions of suffering, becomes a challenge to which there is no easy solution. As a result, some individuals develop a desire to die. The decision to die becomes an active process, as the following quote illustrates:

“Individuals are in this feasible medicine, I'm talking about cancer, the [health professionals] are now also confronted with the feasible death” (FG 5_C1_86).

To follow this path of hastening death, according to the participants, many individuals inform themselves via the internet, other media or talk to other individuals about their situation. They often approach health professionals with their ideas, also about VSED, and ask them to accompany them. Participating health professionals express that they can protect themselves from VSED; they are addressed by the individuals and/or their relatives and must react to it. Most of them have already been addressed. It is therefore important for health professionals to come to terms with the VSED issues. They need to develop a professional stance, at best, before they are confronted with a request to support an individual during VSED. There is no point in closing eyes and hoping not to be approached about it, because VSED is getting better and better known anyway. Even if not every health professional will be confronted with this personally, the discussion about VSED has already arrived in society. Participants criticize that the public debate is very much characterized by journalistic reporting, the brevity of which the phenomenon is not fully explained. As a professional, it is therefore a duty to deal with current issues that affect health in society. However, it is precisely these difficulties that become apparent. Professionals criticize the lack of information themselves. They know too little about the course of VSED and do not feel sufficiently prepared to accompany an individual. Even those who have already accompanied an individual during VSED still have many unanswered questions (about the process in the body; how to deal with delirious individuals who ask for fluids in delirium; what further complications or side effects can be expected,…) and require support. Participants criticize the lack of opportunities for further education and training. Only with comprehensive knowledge is it possible to develop a professional stance. This presupposes that the professionals have dealt with their own values and are aware of them. At the same time, the participants have a contrasting discussion on the extent to which support during VSED is one of the tasks of a health professional. Statements from professional associations and from the legal side are vaguely formulated and lead to uncertainty among professionals. There is also a lack of instructions in the institutions of the participants on how to deal with an individual's death wish through VSED.

### Discussion of the desire to die through VSED

While the participants do not feel responsible for informing an individual about VSED without being asked, it is very important for them to provide information on request. However, first, it must be clarified why an individual refrains from eating and drinking and what reasons are responsible for this behavior. Some participants point out that not every individual who refuses to eat wants to die and not every individual who wants to die through VSED communicates this wish openly and clearly. For this reason, it is important for some participants to first obtain clarity as to whether an individual willing to die is capable of judgment.

“VSED is bound to judgment. Therefore, if someone can no longer [judge] (…), it is out of the question to decide” FG 3_N1_20)

It becomes clear that an individual who lacks decisional capacity will not be supported in their intention. Irrespective of whether an individual openly communicates their wish to die through VSED or whether they tacitly stop eating and drinking, some clarification is needed to clarify further treatment and care. If the reasons for not eating and drinking are not openly communicated, at best, the individuals must be approached to explore the reasons. While a participating ethicist countered that not everyone can talk about death and decisions at the end of life, a participating nurse countered that accompanying a VSED case is tied to conversation. If a direct approach is not possible, health professionals face a great challenge. There is a lack of assessment tools to test the judgment of individuals who are restricted in their communication, but there is also great uncertainty as to whether treatable causes underlie food refusal. If the uncertainties are too great or if an individual is not able to judge, voluntariness cannot be proven, which means that VSED cannot be assumed. The individual must then be treated in the context of her or his illness (e.g., dementia, depression).

If the individual who is willing to die has decisional capacity, the participants agree that the individual has a right to be informed about the VSED. Only through detailed knowledge can an informed decision be made. The problem here is that the treatment of individuals who want to die through VSED is not regulated in Switzerland, and health professionals are expected to make decisions without a legal basis, which is described by the participants as a particular challenge. In addition, it is criticized the topic of VSED is not part of the training for health professions. As a consequence, professionals lack comprehensive knowledge and do not feel sufficiently competent to advise individuals and their relatives. In addition, there is no institution or neutral advice center to which individuals can go. Whether an individual receives counseling depends entirely on whether the health professional has already dealt with the subject. The decision to follow the VSED path cannot be made by the individual who is willing to die alone because he or she is dependent on professional support and the accompaniment of relatives during the course of VSED.

The health professional, who has been asked by the individual willing to die, must now discuss the situation within the team to clarify a decision regarding the accompaniment—yes or no. Participants underline that the team here is not limited to one institution or profession, all professionals must be involved in the discussion, who are necessary to ensure professional accompaniment. The participants argue that the decision cannot be made by one professional alone; the team must decide uniformly for or against an accompaniment. It cannot be ruled out that individual team members will decide against the accompaniment, but the majority will support it. Participants affirm that the attitude of the individual must not be condemned by the team but must be accepted. In this case, it is also conceivable to offer a compromise solution in which the professional who is against accompaniment is not involved in the care of the individual. It is important, however, that those who decide to go for the accompaniment coordinate the process with one another. It is also necessary for one professional to manage the case and be there as contact for the relatives.

*“When it [VSED] takes place at home, there are truly many people involved. Not only the outpatient care, the family physician, and the relatives, but truly quite different ones and that is why I think what would be important is truly: You have to organize yourself at that moment and agree on that and truly say who has the lead. In addition, whoever has the lead then shouts «Attention - watch out, something is not going well. Come to each other!»” (FG 4_N5_87)*

Not only the HCP but also the relatives and all other people involved in accompanying the individual who is willing to die, such as volunteers and neighbors, must be involved in the decision-making process. In most cases, the individual willing to die takes their relatives into their confidence. If this step has not yet been taken, nurses or physicians usually take over this task and become the mediator for the individual who is willing to die. The culture of communication lived within the family has a decisive influence on the willingness to engage in such a conversation. If open communication is lived in the family, topics such as dying or the desire to die can also be addressed more easily than in a family in which many topics are kept silent.

For the decision-making process, the best solution for all participants is to gather all the people involved together at a round table and discuss the situation.

*“Everyone will be informed again. This is a roundtable discussion in which all participants: doctor, nurses, relatives, the individual and external people involved who want to help. Basically, such a conversation and there the individual can express himself as he sees it (...) and then one can decide.” (FG 1_M2_64)*

In this conversation, it is important that all participants have their say that everyone can express their fears and worries. In addition, it is the task of the professionals to inform everyone about the VSED, to describe its course and duration, to explain the processes in the body and to deal with possible side effects such as delirium.

### The silent path

Some participants reported that not all food refusals were necessarily questioned but were silently accepted. In these cases, the participants interpreted food renunciation as a natural dying process without further examination of the individual. It is striking that in these cases, very old, frail, and incapable individuals were always reported, but not young and healthy individuals. It was stated by some participants that in old age or in a fragile condition, an individual is expected to die naturally. Therefore, reduced food intake is not questioned but automatically interpreted as a natural dying process. A voluntary act is not considered and therefore not checked. In contrast to young or healthy individuals, who are not expected to die in the foreseeable future, the renunciation of food and liquids would be questioned.

In individuals with cognitive impairments and inability to judge, it can be observed that some participants rely on their intuition and do not always verify it. They claim to know through gestures and facial expressions that an individual no longer wants to eat or drink.

*“No, consciously it's not. I think it is like in a process to take an unconscious farewell. Because the basic need to eat, everyone has that. And when you stop eating, you know that life is coming to an end. (...) And if she doesn't want to eat any more, then you have to accept that, too” (FG 1_N4_67).*

## Discussion

This study aimed to explore how HCP in Switzerland accompany individuals during VSED and to analyze this decision-making process. The main results of this study showed that VSED has become an influential topic in the Swiss health care system and that, depending on the attitudes of the participants, two very different behavior patterns are used to address it.

### VSED has achieved social recognition

While silence about death has been maintained in recent decades, a social change towards openness to talk about dying and death is becoming apparent. Through medical progress, the dying process can be delayed, but it leads to those affected becoming aware of the presence of dying and actively integrating dying into their lives [71]. This can be seen in the fact that an increasing number of individuals in different contexts, talking to nurses, doctors, counseling centers and pastors feel the need to talk about death and to decide on their dying process [72]. Individual's interest has led to the fact that the once forgotten possibility of hastening death through VSED, which has been known and practiced since ancient times [2, 73], has now re-established itself as a viable end of life option of individuals in Switzerland.

### Participants feel insufficiently prepared

Participants unanimously expressed they felt ill-prepared for the care of an individual during VSED. Participants working as nurses, physicians or as counselors lack knowledge about the course of VSED and what has to be observed. There are also no guidelines in Switzerland that can be followed, as is already the case in the Netherlands [74], for example. Participants expressed a need to be fully informed about how to deal with VSED and that the institutions establish clear rules on whether or not an individual may be accompanied during VSED. Only by consciously dealing with the topic can health professionals develop a professional stance. Some of them will generally oppose support during VSED, which should be accepted by the team and the institution. If a VSED case is treated, this professional should be explicitly assigned to other individuals. Others decide on a VSED accompaniment, which can then be checked depending on the case.

### Unequal handling of VSED cases

The analyses reveal two behavior patterns, the discussed path and the silent path, for dealing with individuals who voluntarily refrain from eating and drinking. Certainly, it is different to consider whether an individual communicates his or her wish to die openly or whether he or she carries it out tacitly and secretly [41]. However, a systematic approach or assessment to food refusal should be uniform in all cases.

The discussed path, shows that experience in accompanying individuals during VSED has already been gained in individual institutions and that own concepts have been developed on how adequate handling can and should be designed. Some of the recommendations given have already been implemented, others merely demanded. For systematically regulated monitoring, a summary of the experiences from the institutions and the findings from research is recommended.

In the silent path, HCP declare that they rely on their intuition instead of forcing a standardized examination of the individual's eating habits. It is known that intuition is more than just a “gut feeling.” It is rather a combination of knowledge and care experience [75]. It is doubtful, however, to rely solely on intuition, even though nurses have in any case gathered little knowledge about VSED or about the unspoken or concealed form of VSED.

### Implementation in practice and research

The results of this study show that a professional approach to the subject is essential but that knowledge about the VSED is still insufficient. In this respect, it is recommended that further training on the current state of knowledge be offered to all those involved in the health care system and that a guideline be developed in parallel to this to ensure a basis for systematic care.

### Strengths and limitations

The research results show benefits for both practice and research. The quality of the results was achieved by following the analytical steps and their documentation, as well as by validation from another researcher. A high degree of credibility of the results can be assumed, since the topic was already known to the participants before the palliative care conference and the participants explicitly registered for it. In addition, a lecture on the current state of knowledge was given before the focus group interviews so that no misunderstandings about what VSED was to be expected. However, the recruitment strategy of including participants in an expert meeting can also influence the data, as participation in the meeting already allows a strong view or interest in VSED. Through the exchange within the focus groups, all points of view could be placed, and counter positions could be taken.

Due to the recruitment strategy, theoretical sampling was not possible, which also meant that a check for theoretical saturation was not possible. However, due to the large sample size and the heterogeneity in the sample as a whole and within the focus groups, it can be assumed that theoretical saturation had been reached, although it must be constrained that only one relative and two physicians could be included in the study. Nevertheless, the authors assume that the results are valid. This is the first focus group study on the VSED using the grounded theory approach. With this approach, previously unknown findings could be identified, which leads to a gain of knowledge in the care of individuals at the end of life. It is recommended to conduct further qualitative studies with the HCP. In particular, observational studies in long-term care institutions on nutritional behavior are necessary to explore the implicit VSED in more detail. Interviews with individuals themselves and their relatives are also important to understand their needs, motivations, burdens, and experiences.

## Conclusion

This study shows that VSED has become a relevant issue for the HCP in end-of-life care, which has a direct impact on the Swiss health care system. To date, however, no procedures have been laid down in Switzerland to deal with this issue, either systematically or on a case-by-case basis. There is a lack of regulations within institutions and a lack of knowledge at the professional level. One of the consequences of this is that individuals who express a wish to die through the VSED or who abstain from food without expressing it are treated very individually. This individual care involves risks. On the one hand, there are uncertainties as to whether the individuals may be treated in the institution, what options are available to professionals and what the best possible care actually looks like. On the other hand, it can lead to the fact that individuals who refuse to eat tacitly are interpreted as individuals in the dying process, although this is not the case. It is therefore possible that individuals die who could have been helped by therapy.

## Data Availability

The datasets used and/or analysed during the current study are available from the corresponding author on reasonable request and after relevant ethical review board approval.
